# Exploratory cluster analysis of noninvasive vascular measures reveals heterogeneous vascular profiles after stroke

**DOI:** 10.1371/journal.pone.0362305

**Published:** 2026-11-06

**Authors:** Bria L. Bartsch, Amanda N. Engler, Sandra A. Billinger

**Affiliations:** 1 Department of Neurology, University of Kansas Medical Center, Kansas City, Kansas, United States of America; 2 Department of Physical Therapy, Rehabilitation Science, and Athletic Training, University of Kansas Medical Center, Kansas City, Kansas, United States of America; 3 Department of Neurology, University of Kansas Alzheimer’s Disease Research Center, Fairway, Kansas, United States of America; 4 Department of Physical Medicine and Rehabilitation, University of Kansas Medical Center, Kansas City, Kansas, United States of America; 5 Department of Cell Biology and Physiology, University of Kansas Medical Center, Kansas City, Kansas, United States of America; University of Messina, ITALY

## Abstract

Vascular dysfunction is prevalent after stroke and contributes to elevated cardiovascular risk; however, the extent to which vascular heterogeneity exists in this population remains unclear. This study examined whether distinct multi-domain cluster-derived vascular profiles are present in individuals with chronic stroke and whether these profiles are associated with functional performance. Vascular structure and function were assessed using flow-mediated dilation, carotid intima-media thickness, pulse wave velocity, and augmentation index, with age included in vascular clustering to account for vascular aging. Unsupervised k-means clustering was applied to identify vascular profiles. Functional performance was assessed using gait speed and predicted peak oxygen consumption (VO_2peak_) derived from a submaximal exercise test. Between-cluster differences were characterized using analysis of variance. Forty-five individuals with chronic stroke (62 ± 12 years; 29 ± 30 months post-stroke) were included. Three vascular profiles were identified (cluster 1: n = 13; cluster 2: n = 11; cluster 3: n = 21), with significant differences across clusters for all vascular measures (p < 0.01). Although age increased across clusters, vascular structure and function did not change uniformly. One profile exhibited elevated arterial wave reflection despite relatively preserved endothelial function and arterial structure, whereas another demonstrated impairment in all vascular variables. Vascular profile was not associated with predicted VO_2peak_ (p = 0.31) or gait speed (p = 0.65). Three exploratory cluster-derived vascular profiles emerged among participants with chronic stroke, and age did not fully account for the observed vascular heterogeneity. Multi-domain cluster-derived vascular profiling revealed heterogeneous vascular profiles in individuals with chronic stroke, providing a basis for future studies to evaluate their relevance to cardiovascular risk stratification and long-term health management. NCT05936008: https://clinicaltrials.gov/study/NCT05936008

## Introduction

Stroke is commonly preceded by systemic vascular dysfunction which persists into the chronic phase of recovery.[1,2] Specifically, individuals with stroke often exhibit impaired vascular endothelial function and cerebrovascular hemodynamics, increased intima-media thickness, and elevated arterial stiffness.[2–7] These vascular factors negatively impact functional capacity and cardiovascular risk and likely, recovery potential.[8–10]

Despite the presence of vascular dysfunction being reported post-stroke, [2–7] vascular heterogeneity in this population remains under explored. Individuals across the spectrum of stroke recovery exhibit notable variations in peripheral and central vascular function.[2,4,6,11] Thus, developing an understanding of the vascular profiles which exist in individuals with stroke could help elucidate which patients may benefit from targeted vascular interventions.

In cardiovascular and aging populations, vascular phenotyping has become a valuable technique to further knowledge regarding the vascular profiles which exist in disease states and promote medical intervention prior to cardiovascular event onset.[12–15] Common vascular variables explored include flow-mediated dilation (FMD), peripheral artery tonometry, pulse wave analysis, pulse wave velocity (PWV), carotid intima-media thickness (IMT), and age.[12–15] However, vascular phenotyping has not been extended to individuals with stroke who may benefit from such characterization due to their elevated risk for subsequent cardiovascular events.

The purpose of this study was to identify multi-domain cluster-derived vascular profiles in individuals with chronic stroke using established noninvasive measures of vascular health. A secondary and exploratory objective was to identify if these vascular profiles correspond to differences in cardiorespiratory fitness or physical function which could inform targeted rehabilitation practice. We hypothesized that distinct cluster-derived vascular profiles would emerge based on systemic measures of vascular endothelial function (FMD), arterial structure (carotid IMT), arterial stiffness (PWV) and wave reflection (augmentation index [AIx]), and age. We further hypothesized that as vascular health decreased by cluster, cardiorespiratory fitness (predicted VO_2peak_) and physical function, as assessed by gait speed (10-meter walk test [10MWT]), would also decline. Identifying the multidomain cluster-derived vascular profiles which exist post-stroke may inform and improve vascular risk stratification, rehabilitation, and long-term health in individuals living with stroke.

## Materials and methods

This is an analysis of baseline data collected as part of a preliminary efficacy trial, Fitness after Stroke (FAST; NCT05936008), conducted between 03/07/2023-08/12/2025.[16] The University of Kansas Medical Center Institutional Review Board approved all study procedures (IRB #00147598). Written informed consent was obtained by all individuals prior to study participation, and procedures complied with the Declaration of Helsinki.

### Participants

Participants were 20–85 years of age, and 6 months to 15 years post-stroke. Participants were able to walk overground without continuous physical assistance, exercise continuously for 3 minutes at 30 watts, able to communicate with investigators, follow a 2-step command and correctly answer consent comprehension questions, participating in <150 minutes of physical activity per week, on stable blood pressure and statin medications for ≥30 days, and had no aerobic exercise contraindications. Participants were excluded if they: 1) Were hospitalized for a cardiac or pulmonary condition in the past 3 months, 2) Had an implanted pacemaker or defibrillator that would limit exercise performance, 3) Reported pain that limits or interferes with activities of daily living or physical activity, 4) Had lower extremity spasticity (Modified Ashworth >2), 5) Had illicit drug use, alcohol abuse, or a diagnosis of significant mental illness in the past 3 months, 6) Reported major post-stroke depression (Patient Health Questionnaire score ≥ 10), 7) Were currently participating in physical therapy focused on lower extremity function or another interventional study that may influence outcomes, 8) Had neurologic, orthopedic, or peripheral vascular conditions that would limit exercise participation, 9) Had oxygen-dependent chronic obstructive pulmonary disease, 10) Were diagnosed with another neurologic condition, or 11) Self-reported pregnancy.

### Vascular endothelial function

Vascular endothelial function was assessed via FMD conducted by a trained research assistant (intra-rater reliability using coefficient of variation: 7.32%) in accordance with current guidelines.[10] Procedures were conducted between 7:00–9:00am to account for diurnal variation in vascular function. Participants were asked to refrain from food or tobacco use for ≥6 hours, alcohol or caffeine for ≥12 hours, and vigorous activity for ≥24 hours. Participants were also asked to withhold anti-hypertensive or statin medications until after the assessments due to the effects that these medications have on vascular function. Adherence to instructions were documented.

For FMD procedures, participants rested in supine for 15 minutes, followed by a 1-minute continuous baseline recording of the brachial artery diameter and blood flow velocity (insonation angle ≤60º; GE Healthcare LOGIQ Ultrasound, Chicago, IL). Next, a rapid inflation pneumatic cuff, 1–2 cm distal to the antecubital fossa, was inflated to 220 mmHg for 5 minutes. Following cuff deflation, the brachial artery and blood flow velocity were continuously recorded for 3 minutes. Electrocardiogram gating was used throughout image acquisition to minimize the influence of arterial compliance on vascular endothelial function. Procedures were completed bilaterally, with the stroke-affected upper extremity manually stabilized as necessary. Recordings were analyzed offline using semi-automated edge-detection software (Brachial Analyzer, Medical Imaging Applications, Coralville, Iowa), to reduce investigator bias.[10,17–19] %FMD was determined as the ([peak diameter-baseline diameter]/baseline diameter)*100.

### Arterial structure

Arterial structure was assessed using carotid artery intima media thickness by a trained research assistant. With the participant in supine, 30-second recordings of the bilateral common carotid arteries were obtained ~1 inch distal to the carotid bulb using an 11MHz probe (GE Healthcare LOGIQ Ultrasound, Chicago, IL). Intima-media thickness was determined offline using semi-automated edge detection software (Carotid Analyzer, Medical Imaging Applications, Coralville, Iowa).

### Large artery stiffness and wave reflection

Large artery stiffness and wave reflection were assessed with participants in the supine position using PWV and AIx, respectively (SphygmoCor, Itasca, IL). For PWV, a cuff was placed around the participant’s upper thigh, and the carotid and femoral pulses were located via palpation. The distance between the carotid pulse and suprasternal notch, suprasternal notch and thigh cuff, and femoral pulse and thigh cuff were measured and recorded. The femoral and carotid pulse waves were then recorded simultaneously, and PWV, auto-calculated using PWV (m/s) = PWV distance/pulse transit time where PWV distance was calculated using the subtraction method. AIx was derived using pulse wave analysis with a blood pressure cuff over the brachial artery. AIx was auto-calculated using a transfer function applied to the brachial waveform to estimate the central aortic pressure waveform, and AIx = (Augmentation pressure/pulse pressure)*100. For both PWV and AIx, two recordings were obtained ~1-minute apart and averaged.

### Functional performance measures

Cardiorespiratory fitness was determined using the Total Body Recumbent Stepper (TBRS) submaximal exercise test, [20] which is reliable for predicting VO_2peak_ in individuals with stroke.[21,22] Detailed methodology for the TBRS submaximal exercise test have been published elsewhere, [20–22] but in brief, participants progressed through up to 4 stages of increasing workload until either the test was complete, participants reached 85% of their age-predicted heart rate maximum, or requested to stop. VO_2peak_ was then predicted using the following equation: VO_2peak_ = 125.707 - 0.476*Age + 7.686*Sex – 0.451*Weight + 0.179*Work – 0.415*Heart Rate where age is in years, weight in kilograms, work in watts, heart rate in beats per minute, and a 1 is assigned for males versus 0 for females.

Gait speed was determined using the 10MWT, where participants were instructed to walk at their normal, comfortable speed over a 14-meter path where the middle 10-meters were timed. Two trials were completed and averaged. Participants were allowed to use assistive and orthotic devices, as necessary.

### Statistical analysis

Unsupervised machine-learning clustering was applied to vascular measures (FMD, IMT, AIx, PWV) with age included to determine vascular profiles. Prior to clustering, variables were converted to Z-scores to remove the influence of measurement units on cluster assignment. Using paired samples t-tests, we determined that neither FMD (p = 0.35) nor IMT (p = 0.70) significantly differed by stroke-affected side, and as such bilateral FMD and IMT variables were each averaged. K-means clustering was then used to identify exploratory vascular profiles, and the candidate cluster solutions were determined using elbow (within-cluster sum of squares) and silhouette methods. K-means clustering was performed with 25 random starts to improve stability, and robustness of the three-cluster solution was evaluated using bootstrap resampling, sensitivity analyses with multivariate outlier exclusion, and hierarchical clustering with Ward’s linkage. Additionally, age is a known influencer of vascular structure and function, and medication use may influence AIx. As such, sensitivity analyses excluding age and AIx from the clustering variables were performed to evaluate cluster structure stability independent of age and AIx.

Cluster characteristics were summarized using mean (standard deviation) for all variables. Between-cluster differences in the vascular variables were assessed using one-way analyses of variance (ANOVAs) with Tukey post-hoc comparisons. ANOVA results are presented to characterize differences among the clusters rather than to independently validate them, as the same vascular variables were used to derive the clusters. Effect sizes are reported as eta squared (η²), and pairwise mean differences are reported with 95% confidence intervals. To compare functional measures of cardiorespiratory fitness (predicted VO_2peak_) and comfortable gait speed between clusters, one-way ANOVAs with Tukey post-hoc comparisons were used. Analyses were conducted using R version 4.2.2, and significance was set at alpha = 0.05. Values are reported as mean (standard deviation) unless otherwise specified.

## Results

Forty-five out of 50 participants with complete baseline data sets were included in the analysis. PWV measurements could not be obtained in five participants despite repeated acquisition attempts. Four of these participants had obesity (BMI 35.19–45.73 kg/m²), which may affect pulse wave acquisition and signal quality, and one participant had a complex cardiovascular history resulting in an unobtainable waveform. Participants were 62.18 (12.24) years of age and 29.22 (30.33) months post-stroke. Demographics are reported in Table 1.

**Table 1:** Participant demographics.

	Cluster 1 (n = 13)	Cluster 2 (n = 11)	Cluster 3 (n = 21)	All (n = 45)
**Age**	48.08 (7.97)	62.82 (9.65)	70.57 (6.45)	62.18 (12.24)
**Sex, female (%)**	6 (46.20)	5 (45.50)	10 (47.60)	21 (46.70)
**Race, n (%)** **White/Caucasian** **Black/African American** **Asian** **American Indian/Alaska** **Native**	9 (69.23) 4 (30.77) 0 (0.00) 0 (0.00)	8 (72.73) 3 (27.27) 0 (0.00) 1 (9.09)	15 (71.43) 6 (28.57) 0 (0.00) 0 (0.00)	32 (71.11) 13 (28.89) 0 (0.00) 1 (2.22)
**Ethnicity, n (%)** **Hispanic** **Non-Hispanic**	0 (0.00) 13 (100.00)	0 (0.00) 11 (100.00)	0 (0.00) 21 (100.00)	0 (0.00) 45 (100.00)
**BMI (kg/m**^**2**^)	30.26 (5.12)	29.61 (7.63)	30.05 (7.53)	30.00 (6.80)
**Time Post-Stroke (months)**	29.39 (29.49)	32.09 (33.21)	27.61 (30.72)	29.22 (30.33)
**Type of stroke, ischemic (%)**	9 (69.23)	7 (63.64)	16 (76.19)	32 (71.11)
**Stroke Location, n (%)** **Right cortical** **Left cortical** **Right subcortical** **Left subcortical** **Bilateral** **Cerebellar** **Brainstem**	5 (38.46) 4 (30.77) 0 (0.00) 2 (15.38) 0 (0.00) 0 (0.00) 2 (15.38)	4 (36.36) 4 (36.36) 2 (18.18) 2 (18.18) 0 (0.00) 0 (0.00) 0 (0.00)	6 (28.57) 7 (33.33) 1 (4.76) 2 (9.52) 2 (9.52) 2 (9.52) 2 (9.52)	15 (33.33) 15 (33.33) 3 (6.67) 6 (13.33) 2 (4.44) 2 (4.44) 4 (8.89)
**Systolic BP (mmHg)**	119.15 (18.33)	122.50 (12.29)	139.52 (23.14)	129.64 (21.62)
**Diastolic BP (mmHg)**	76.46 (12.96)	74.20 (10.92)	77.90 (14.43)	76.64 (13.07)
**Resting HR (bpm)**	62.69 (11.29)	59.77 (13.63)	59.40 (7.63)	60.44 (10.28)
**Smoking status, n (%)**	0 (0.00)	1 (9.09)	2 (9.52)	3 (6.67)
**Hypertension, n (%)**	9 (69.23)	8 (72.73)	19 (90.58)	36 (80.00)
**Dyslipidemia, n (%)**	10 (76.92)	10 (90.91)	20 (95.24)	40 (88.89)
**Hyperglycemia, n (%)**	3 (23.08)	3 (27.27)	9 (42.86)	15 (33.33)

BMI, body mass index; kg/m^2^, kilogram per square meter; BP, blood pressure; mmHg, millimeters of mercury; HR, heart rate; bpm, beats per minute; 1 participant reported multiple races; Smoking status indicates participants who are either current smokers or have smoked in the previous 6 months

Elbow and silhouette analyses revealed modest support for k = 2−4 clustering solutions, with average silhouette widths between 0.23–0.28 (S1 Fig). Improvement in within-cluster sum of squares diminished beyond 3 clusters, and as such, an exploratory 3-cluster solution was used. Bootstrap resampling indicated moderate cluster stability (mean adjusted Rand index = 0.57), while hierarchical clustering via Ward’s linkage indicated moderate agreement with the k-means solution (adjusted Rand index = 0.59). No influential multivariate outliers were identified.

### Cluster-derived vascular profiles

We identified 3 vascular profiles using exploratory K-means clustering in individuals with chronic stroke (Table 2). Significant effects of vascular profile were observed for all included variables (Fig 1): FMD (F(2,42) = 6.47, **p < 0.01,** η² = 0.24), carotid IMT (F(2,42) = 15.21, **p < 0.001,** η² = 0.42), AIx (F(2,42) = 19.84, **p < 0.001,** η² = 0.49), PWV (F(2,42) = 28.1, **p < 0.001,** η² = 0.57, 95% CI [0.40, 1.00]), and age (F(2,42) = 33.83, **p < 0.001,** η² = 0.62). S1 Table provides additional information regarding between cluster comparisons.

**Table 2:** Cluster-derived vascular profiles.

Cluster	N	FMD	IMT	AIx	PWV	Age
**1**	13	5.84 (2.78)	0.66 (0.10)	24.77 (7.67)	7.75 (0.94)	48.08 (7.97)
**2**	11	6.31 (3.20)	0.71 (0.06)	45.68 (7.40)	8.79 (1.12)	62.82 (9.65)
**3**	21	3.34 (1.98)	0.91 (0.19)	35.31 (8.71)	10.95 (1.28)	70.57 (6.45)

FMD, flow-mediated dilation; IMT, intima-media thickness; AIx, augmentation index; PWV, pulse-wave velocity

**Fig 1:**
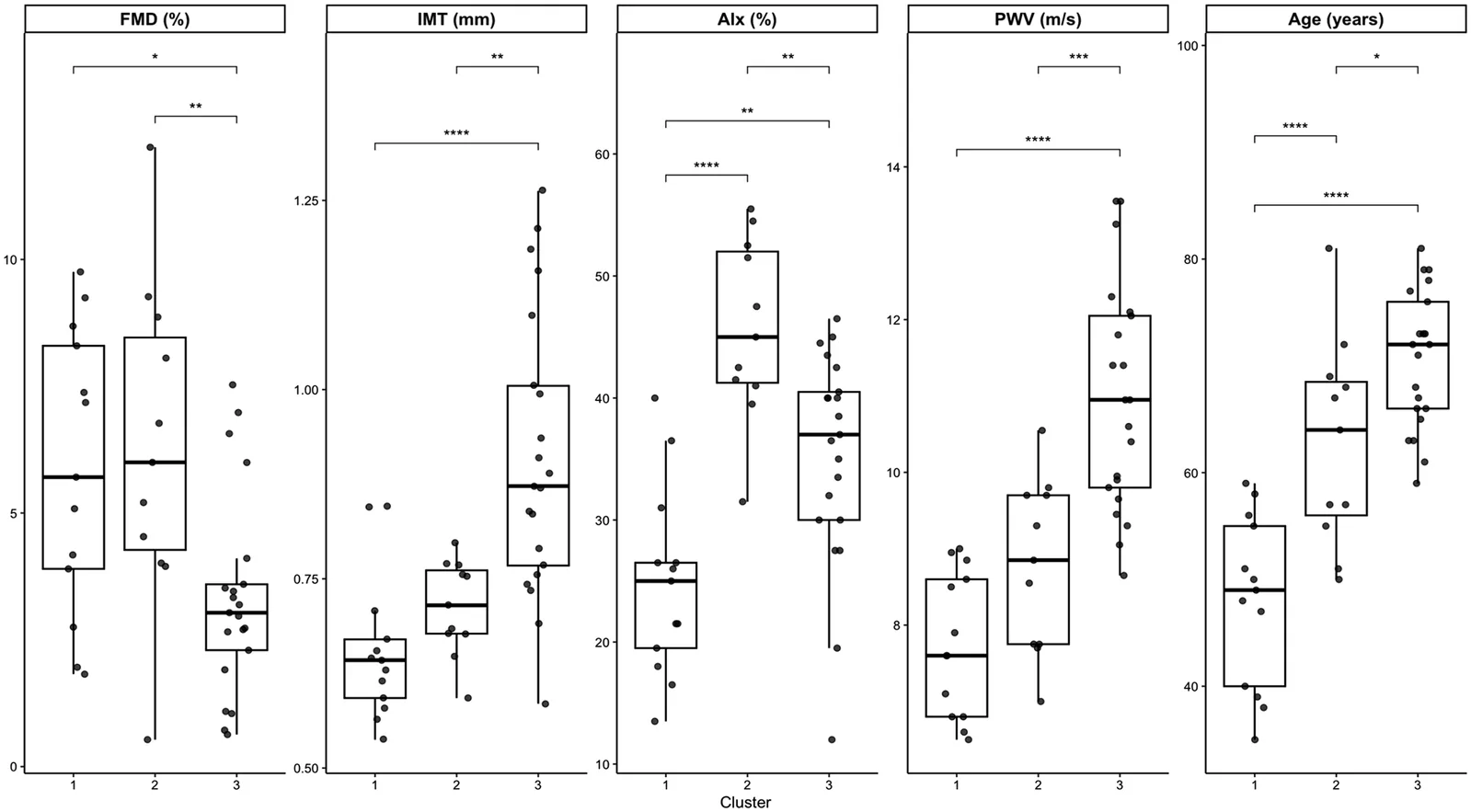
Vascular measures by clusters. FMD, flow-mediated dilation; IMT, intima-media thickness; mm, millimeters; AIx, augmentation index; PWV, pulse-wave velocity; m/s, meters per second. *p < 0.05, **p < 0.01, ***p < 0.001, ****p < 0.0001.

Post-hoc analyses revealed a significant increase in age from profile 1–3 (p < 0.05), consistent with a vascular aging profile. Vascular endothelial function (FMD), carotid arterial structure (IMT), and central arterial stiffness (PWV) did not differ between clusters 1 and 2 (p > 0.05). However, cluster 2 demonstrated the highest arterial wave reflection (AIx) amongst all groups (p < 0.01), suggesting an increase in arterial wave reflection despite preserved vascular endothelial function and no significant changes in PWV from cluster 1–2. Cluster 3 demonstrated the lowest vascular endothelial function (FMD; p < 0.01), thickest carotid IMT (p < 0.01), and greatest central arterial stiffness (PWV; p < 0.001), reflecting a more adverse vascular function and structure profile among older individuals post-stroke.

Given the unexpected finding of cluster 2 having a significantly higher wave reflection (AIx) than cluster 3 despite a younger mean age and relatively preserved arterial structure and function, we explored antihypertensive medication use between clusters (Table 3). In cluster 1, 9 (69.23%) participants were taking antihypertensive medication, including 3 (23.08%) taking a beta-blocker and 3 (23.08%) taking more than 1 antihypertensive medication. In cluster 2, 7 (63.64%) participants were taking antihypertensive medication, including 1 (9.09%) participant taking a beta-blocker and 2 (18.18%) taking more than 1 antihypertensive medication. In cluster 3, 18 (85.71%) participants were taking antihypertensive medication, including 10 (47.62%) taking a beta-blocker and 12 (57.14%) taking more than 1 antihypertensive medication. Thus, the greater prevalence of antihypertensive medication use in cluster 3 compared to cluster 2 may have contributed to the differences observed in arterial wave reflection.

**Table 3:** Medication use across clusters.

Medication	Cluster 1 (n = 13)	Cluster 2 (n = 11)	Cluster 3 (n = 21)
**Antihypertensive Medication**			
Aldactone	0	0	1
Amlodipine	2	4	7
Carvedilol	2	1	4
Chlorthalidone	1	0	1
Diltiazem	0	0	1
Doxazosin	0	0	1
Furosemide	1	0	1
Hydralazine	0	0	1
Hydrochlorothiazide	2	1	1
Lisinopril	3	2	2
Losartan	2	2	8
Metoprolol	1	0	5
Nebivolol	0	0	1
Olmesartan	1	0	0
Valsartan	0	0	1
**Statin/Cholesterol Medication**			
Atorvastatin	5	7	12
Evolocumab	1	0	0
Fenofibrate	0	1	1
Lovastatin	0	0	1
Rosuvastatin	4	1	6
**Antiplatelet/Anticoagulant Medication**			
Aspirin	6	3	12
Clopidogrel	1	2	4
Eliquis	2	0	5
Enoxaparin	0	1	0

### Sensitivity analyses

Given the known influence of age on vascular function [23], we performed a sensitivity analysis with age removed from the clustering model; k was retained at 3 for comparison with the primary analysis. Following the removal of age, the clustering inputs produced appreciable changes in cluster membership (adjusted Rand index = 0.53). Cluster sizes were 16, 13, and 16 participants for clusters 1, 2, and 3, respectively. Corresponding centroids (mean FMD, IMT, AIx, and PWV) were 5.24%, 0.70 mm, 22.88%, and 8.33 m/s for cluster 1; 6.28%, 0.71 mm, 45.69%, and 8.67 m/s for cluster 2; and 3.12%, 0.94 mm, 37.88%, and 11.35 m/s for cluster 3. Descriptive ANOVAs characterized differences among these profiles in FMD (p < 0.01), IMT (p < 0.001), AIx (p < 0.001), and PWV (p < 0.001). As such, heterogenous vascular profiles remained, suggesting that age did not fully account for the observed vascular heterogeneity.

Given the potential influence of blood pressure medication on AIx, we conducted an analysis with AIx removed from the cluster model (Fig 2). This resulted in low-to-moderate cluster agreement between the AIx-removed model and original (adjusted Rand index = 0.39), indicating that cluster membership was heavily influenced by AIx. Significant between-cluster differences remained for IMT (p < 0.001), PWV (p < 0.001), and age (p < 0.001). However, between-cluster differences in FMD were no longer significant (p = 0.10). As such, findings suggest that AIx provided a meaningful contribution to the original clustering model, although patterns of vascular dysfunction persisted without the inclusion of AIx.

**Fig 2:**
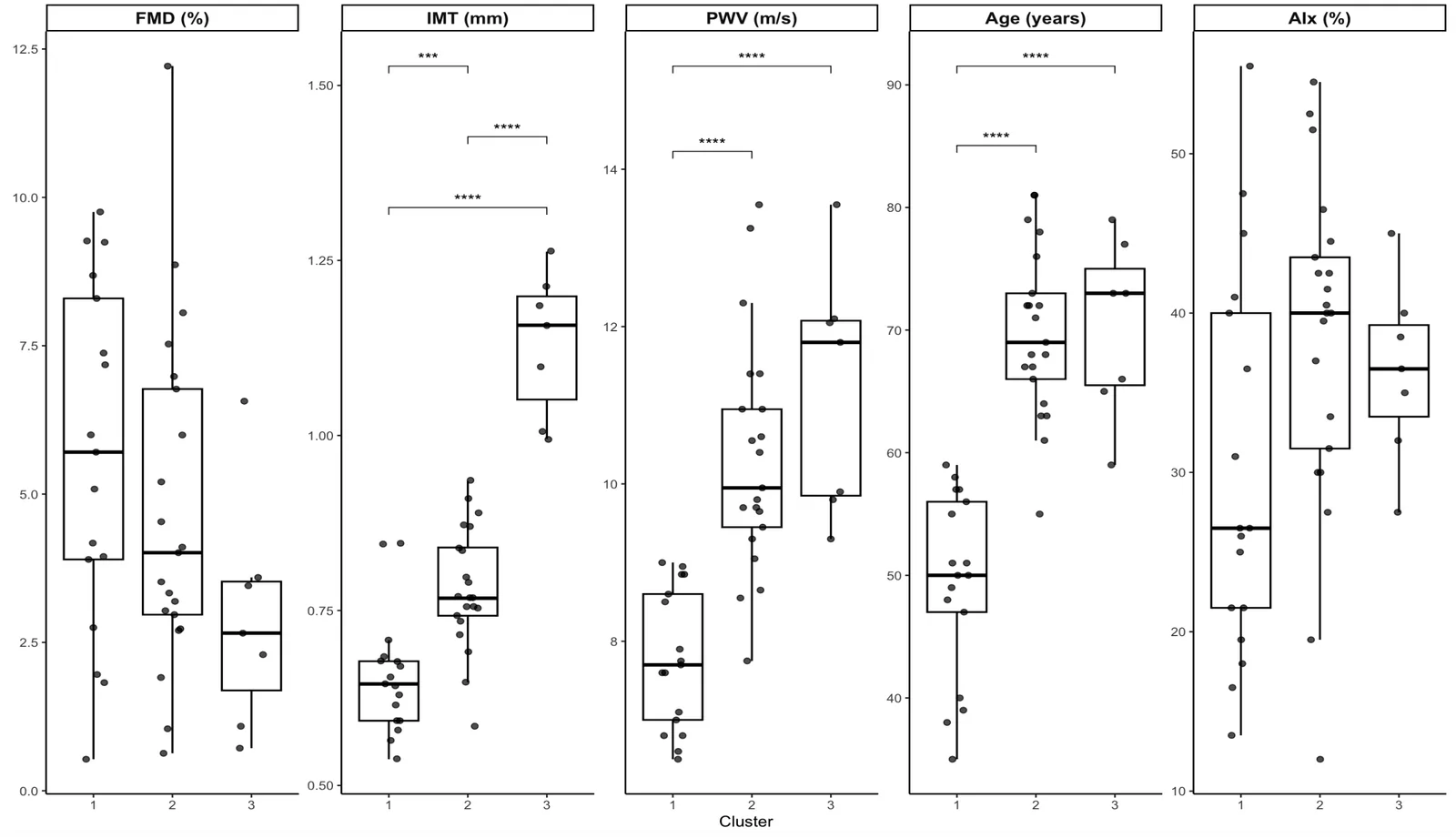
Cluster-derived profiles with AIx removed from model. AIx was not included in cluster generation but is displayed for descriptive comparison across clusters; FMD, flow-mediated dilation; IMT, intima-media thickness; mm, millimeters; AIx, augmentation index; PWV, pulse-wave velocity; m/s, meters per second. *p < 0.05, **p < 0.01, ***p < 0.001, ****p < 0.0001.

### Vascular profiles and functional performance

Average predicted VO_2peak_ (mL/kg/min) across all participants was 25.60 (8.74). Predicted VO_2peak_ values by cluster were as follows: Cluster 1, 26.94 (11.45); Cluster 2, 28.15 (8.30); Cluster 3: 23.45 (6.63). No significant effect of vascular profile on predicted VO_2peak_ was observed (p = 0.31, η² = 0.06, 95% CI: 0.00–0.21).

Normal gait speed (m/s) across all participants was 0.87 (0.42) and 0.79 (0.45), 0.96 (0.51), 0.87 (0.37) for Clusters 1, 2, and 3, respectively. Similarly, no significant effect of vascular profile on gait speed was observed (p = 0.65, η² = 0.02, 95% CI: 0.00–0.13); however, these findings should be interpreted in the context of substantial within-group variability and small cluster sizes.

## Discussion

Findings indicate that while age is a strong driver of vascular structure and function post-stroke, chronological age alone does not fully explain the various vascular profiles observed in this study, and reliance on single vascular metrics may not reveal clinically relevant hemodynamic profiles. Further, and contrary to our hypothesis, we did not observe significance for vascular profile influencing functional performance in individuals with chronic stroke. These exploratory findings provide a basis for future studies to evaluate whether cluster-derived vascular profiles may offer clinically useful information beyond established risk factors.

### Vascular structure and function

The predominant finding that contrasts with the normative pattern of vascular aging was the elevated AIx observed in cluster 2. Despite being significantly younger than cluster 3, cluster 2 exhibited the highest wave reflection across all groups. AIx is a measure of arterial wave reflection that is influenced by factors including arterial properties, peripheral vascular resistance, and heart rate and is associated with cardiovascular event risk and mortality, [24–26] emphasizing the potential clinical importance of this finding.

Typically, AIx increases with age due to structural and functional arterial remodeling where increased collagen deposition, elastin fragmentation, vascular smooth muscle cell proliferation, perivascular inflammation, and endothelial dysfunction increase arterial stiffness and resistance, resulting in an augmented wave reflection. However, despite being ~8 years older, cluster 3 had a ~ 10% lower AIx. In a sample of 297 participants with coronary artery disease, a 10% increase in AIx was associated with a hazard ratio of 1.28.[27] As such, the lower AIx observed in cluster 3 may be meaningful, and the discrepancy between clusters, potentially influenced by differences in blood pressure medication use between groups. In cluster 2, 64% of participants were taking blood pressure medication with 18% taking more than one compared to 86% and 57%, respectively, in cluster 3. Antihypertensive medications, including ACE inhibitors, calcium channel blockers, alpha blockers, beta-blockers, and angiotensin II antagonists reduce total peripheral resistance by promoting vasodilation.[28,29] Further, combining antihypertensive medications may provide increased benefit when compared to taking one antihypertensive medication alone.[29,30] Thus, cluster 3 may have had less peripheral resistance than cluster 2, resulting in a lower AIx. Interestingly, however, cluster 3 had a higher percentage of participants prescribed beta-blocker medications which commonly reduce heart rate.[31] Elevated AIx is associated with lower heart rates due to increased systolic duration.[32] Despite the greater use of beta-blocker medication in cluster 3 compared to 2, AIx values were lower. Additional factors which may have contributed to the observed discrepancies in AIx between groups include metabolic and inflammatory factors, age, and arterial pressure.[33]

When AIx was removed from the clustering model during our sensitivity analyses, only low-to-moderate agreement remained between the new and original clustering models. Given the potential influence of antihypertensive medication on AIx and our sample size, AIx findings should be regarded as exploratory, and future studies should seek to explore the potential influence of medication use on AIx and how such an interaction may influence vascular profiles post-stroke. Given that elevated blood pressure is common post-stroke, [34] and ~80–85% of individuals with stroke and a history of hypertension are on antihypertensive medication, [35,36] the influence of such medication on AIx may be of importance.

Interestingly, cluster 3 exhibited the greatest impairment in vascular endothelial function, as evidenced by FMD, despite having the highest frequency of antihypertensive medication use. Antihypertensive medications are known to improve nitric oxide bioavailability and reduce oxidative stress, which may enhance vascular endothelial function. However, many antihypertensive medications also act on vascular smooth muscle cells by reducing sympathetic vasoconstriction and calcium-mediated vascular smooth muscle cell contraction, ultimately promoting vasodilation. As such, antihypertensive medications may reduce AIx without improving vascular endothelial function, consistent with the AIx and FMD findings observed in cluster 3. Given the prognostic association between endothelial function and cardiovascular events, further work is needed to determine whether endothelial dysfunction represents a modifiable target following stroke. [2] This is further emphasized by all clusters exhibiting an FMD value below 6.5%, which is considered the cutoff for optimal endothelial function.[37] These findings support those previously observed in a systematic review and meta-analysis of 28 studies in which individuals with stroke across the spectrum of recovery exhibited significantly impaired vascular endothelial function.[2]

PWV was not statistically significant between clusters 1 and 2, yet cluster 2 demonstrated a clinically meaningful increase in PWV of ~1 m/s. When compared with previous findings, such an increase may be associated with a 12% increase in cardiovascular event risk, [38] and is similar to that observed in aging populations whereby a 6–8% increase in PWV is observed with every 10 years of aging.[39] The 24.6% increase in PWV observed between clusters 2 and 3 (~7.8 year age difference), however, was much larger than that expected due to normative arterial aging and places individuals in cluster 3 above the 10 m/s cutoff for PWV and cardiovascular event risk.[40] Mechanistically, this greater arterial stiffening may be due to an increased inflammatory state promoting vascular smooth muscle cell proliferation and migration.[41] Supporting this, cluster 3 had the highest IMT across all groups. IMT thickness is primarily driven by the arterial media layer, comprised mainly of vascular smooth muscle cells. These findings echo those observed in a carotid artery phenotype study [15] in which an elevated PWV was observed in participants with carotid hypertrophy.

Our findings for cluster-derived vascular profiles post-stroke are similar to that observed in early hypertension in which the oldest group of participants, although ~28 years younger than ours, exhibited the highest PWV, thickest carotid IMT, and lowest FMD.[12] In contrast to our findings, however, the oldest group with early hypertension also exhibited the highest AIx. These differing findings may be due to medication use, where individuals in the early hypertension study were not allowed to be taking antihypertensive medications, [12] whereas our participants were, and this likely reduced downstream arteriole resistance. Authors in the early hypertension study also concluded that increased arterial stiffening across clusters was observed before changes in vascular endothelial function or carotid IMT.[12] These findings align with ours in which vascular endothelial function and IMT did not differ between clusters 1 and 2 despite differences in wave reflection, as assessed by AIx. Interestingly, the difference in wave reflection observed prior to differences in vascular endothelial function contrasts known vascular pathophysiology in which endothelial dysfunction is one of the earliest hallmarks of vascular health decline. This may be explained by endothelial dysfunction already being present at baseline in participants within our and the early hypertension studies, as evidenced by FMD.[12]

### Vascular profile and functional performance

Contrary to our hypothesis, there was no significant effect of vascular profile on cardiorespiratory fitness or normal gait speed. However, given the exploratory nature of this aim, our modest sample size, and wide motoric heterogeneity present among participants, findings should be interpreted with caution. During physical activity, there is an increased demand for oxygen and nutrient delivery to working muscles.[42,43] Thus, the ability of the vasculature to deliver blood flow to active muscles contributes to aerobic capacity and exercise tolerance.[42,43] Supporting this, a systematic review of 33 studies including healthy adults across the lifespan found that macrovascular function, assessed using measures such as FMD, PWV, AIx, and carotid IMT, was negatively associated with muscle strength and function, likely due to the reduced ability of the vasculature to supply working musculature with oxygen and nutrients.[44] Similar findings were observed in a study of 24 older adults in which AIx was inversely, and FMD positively, associated with muscle power.[44,45] Muscle strength and power are important components of cardiorespiratory fitness and gait speed, and in a study of 2,172 older adults, as PWV increased, gait speed decreased independent of demographics, such as age, sex, and race.[46] This is contrary to the results observed in our study in which significant differences in gait speed between clusters were not observed. This difference in findings may be explained by variations in participant characteristics between studies, or the small cluster sizes and neurologic heterogeneity present in our study.[46] Of the 2,172 older adults, only 7.4% had cerebrovascular disease and the participant cohort had a higher average gait speed of 1.34 m/s than our participants despite a similar average PWV of 8.98 m/s.[46] As such gait speed within our participant group may have been limited by hemiparetic gait impairment or cluster size, masking the influence of vascular function on gait speed in our participants with chronic stroke.

Similar to gait speed, no significant effect of vascular profile on predicted VO_2peak_ was observed in our participants with chronic stroke. Previous studies exploring cardiorespiratory fitness in adults with and without disease have documented a relationship between FMD, PWV, AIx, and cardiorespiratory fitness, whereby as vascular function declines, so does fitness.[47,48] The discrepancy between these findings and ours may be due to variations in study methodology where previous studies [47,48] used maximal exercise testing in individuals without cerebrovascular disease versus submaximal exercise testing used in our study. Likewise, there was large within-group variation for predicted VO_2peak_ present in our sample, which may have contributed to our null findings. Additional research has found a mediating effect of body composition, such as percent body fat and visceral adipose tissue mass, on the relationship between macrovascular function and cardiorespiratory fitness.[49] However, we did not collect the data to examine how body composition influenced our findings.

### Limitations

Our study is among the first to explore cluster-derived vascular profiles in individuals with stroke. While our findings are informative and may guide future research, caution should be exercised when interpreting findings as our sample sizes ranged from 11 to 21 between clusters and reflect cluster-derived profiles rather than established clinical phenotypes. Further, the choice of using 3 clusters was exploratory based on within-cluster sum of squares findings and does not represent a validated biological solution. For AIx, we report unnormalized values to characterize augmentation under each participant’s resting physiological conditions; therefore, AIx values should not be interpreted as heart rate-independent measures of intrinsic vascular properties. The exclusion of 5 participants due to missing PWV data may have introduced selection bias. Future research should seek to explore vascular profiles in a larger cohort of individuals with stroke.

While we did not observe a significant effect of vascular profile on physical function, this analysis should be considered exploratory, and additional studies are required to determine the generalizability and reproducibility of these findings. Furthermore, because these analyses were cross-sectional and limited to baseline vascular measures, we were unable to determine whether cluster membership was associated with future clinical outcomes or prognosis. Additionally, medication use may influence the vascular findings observed in our study. However, such findings are informative given the high prevalence of antihypertensive medication use in individuals recovering from stroke. Lastly, this study did not explore if and how type of stroke (ischemic vs. hemorrhagic) or ischemic stroke subtype may influence vascular profiles.

## Conclusion

Findings demonstrate that vascular health post-stroke is heterogeneous and cannot be explained by normative arterial aging alone. While age significantly increased across clusters, vascular structure and function did not change in a uniform manner. Clusters revealed varied pathophysiologic vascular health, likely due to antihypertensive medication use and maladaptive arterial remodeling. Our novel findings of cluster-derived vascular profiles, characterized by altered endothelial function and arterial stiffness, highlight the intricacy of post-stroke vascular health. Although we did not observe significance for vascular profile being associated with functional performance, findings emphasize the importance of evaluating multiple vascular metrics to better characterize post-stroke vascular health. Future research should explore the evolution of vascular profiles over time and seek to identify targeted interventions to improve long-term cardiovascular health.

## Supporting information

S1 Fig Elbow and silhouette analyses. (A) Elbow plot demonstrating within-cluster sum of squares across candidate cluster solutions; (B) Silhouette width across candidate clusters; (C) Distribution of adjusted Rand indices from Bootstrap resampling analyses.
(TIF)

S1 Table Between cluster comparisons. FMD, flow-mediated dilation; IMT, intima-media thickness; mm, millimeters; AIx, augmentation index; PWV, pulse-wave velocity; m, meters; s, second.
(DOCX)
