# Supplementary material for: Exploratory cluster analysis of noninvasive vascular measures reveals heterogeneous vascular profiles after stroke
Source: PLoS One. 2026 Nov 6;21(11):e0362305. doi: 10.1371/journal.pone.0362305 (PMC13742943; doi:10.1371/journal.pone.0362305)
Supplement: Supplementary file 1 [file pone.0362305.s002.docx]

| **Comparison** | **Mean difference** | **95% CI** | **Adjusted p-value** |
| --- | --- | --- | --- |
| **FMD (%)**  2 vs 1  3 vs 1  3 vs 2 | 0.47  -2.51  -2.97 | -2.07 to 3.01  -4.69 to -0.32  -5.28 to -0.67 | 0.90  0.02  < 0.01 |
| **IMT (mm)**  2 vs 1  3 vs 1  3 vs 2 | 0.06  0.25  0.20 | -0.08 to 0.20  0.13 to 0.38  0.07 to 0.33 | 0.60  < 0.001  < 0.01 |
| **AIx (%)**  2 vs 1  3 vs 1  3 vs 2 | 20.91  10.54  -10.37 | 12.83 to 28.99  3.58 to 17.50  -17.71 to -3.03 | < 0.001  < 0.01  < 0.01 |
| **PWV (m/s)**  2 vs 1  3 vs 1  3 vs 2 | 1.03  3.20  2.17 | -0.22 to 2.29  2.12 to 4.28  1.03 to 3.31 | 0.13  < 0.001  < 0.001 |
| **Age (years)**  2 vs 1  3 vs 1  3 vs 2 | 14.74  22.49  7.75 | 7.02 to 22.46  15.85 to 29.14  0.74 to 14.77 | < 0.001  < 0.001  0.03 |

Supplementary Table 1. Between Cluster Comparisons

FMD, flow-mediated dilation; IMT, intima-media thickness; mm, millimeters; AIx, augmentation index; PWV, pulse-wave velocity; m, meters; s, second
